# Suprabasin Is Hypomethylated and Associated with Metastasis in Salivary Adenoid Cystic Carcinoma

**DOI:** 10.1371/journal.pone.0048582

**Published:** 2012-11-07

**Authors:** Chunbo Shao, Marietta Tan, Justin A. Bishop, Jia Liu, Weiliang Bai, Daria A. Gaykalova, Takenori Ogawa, Ami R. Vikani, Yuri Agrawal, Ryan J. Li, Myoung Sook Kim, William H. Westra, David Sidransky, Joseph A. Califano, Patrick K. Ha

**Affiliations:** 1 Department of Otolaryngology-Head and Neck Surgery, the Johns Hopkins Medical Institutions, Baltimore, Maryland, United States of America; 2 Department of Surgical Pathology, the Johns Hopkins Medical Institutions, Baltimore, Maryland, United States of America; 3 University of Pittsburgh School of Medicine, Pittsburgh, Pennsylvania, United States of America; 4 Department of Otorhinolaryngology, Shengjing Hospital, China Medical University, Shenyang, China; 5 Department of Otolaryngology-Head and Neck Surgery, Tohoku University School of Medicine, Sendai, Miyagi, Japan; 6 The George Washington University School of Medicine, Washington D.C., United States of America; 7 Milton J Dance Jr. Head and Neck Center at the Greater Baltimore Medical Center, Baltimore, Maryland, United States of America; Barts & The London School of Medicine and Dentistry, Queen Mary University of London, United Kingdom

## Abstract

**Background:**

Salivary gland adenoid cystic carcinoma (ACC) is a rare cancer, accounting for only 1% of all head and neck malignancies. ACC is well known for perineural invasion and distant metastasis, but its underlying molecular mechanisms of carcinogenesis are still unclear.

**Principal Findings:**

Here, we show that a novel oncogenic candidate, suprabasin (SBSN), plays important roles in maintaining the anchorage-independent and anchorage-dependent cell proliferation in ACC by using SBSN shRNA stably transfected ACC cell line clones. SBSN is also important in maintaining the invasive/metastatic capability in ACC by Matrigel invasion assay. More interestingly, SBSN transcription is significantly upregulated by DNA demethylation induced by 5-aza-2′-deoxycytidine plus trichostatin A treatment and the DNA methylation levels of the SBSN CpG island located in the second intron were validated to be significantly hypomethylated in primary ACC samples versus normal salivary gland tissues.

**Conclusions/Significance:**

Taken together, these results support SBSN as novel oncogene candidate in ACC, and the methylation changes could be a promising biomarker for ACC.

## Introduction

DNA methylation changes, including both hypomethylation and hypermethylation, are commonly found in human cancers [1], [2] including salivary gland adenoid cystic carcinoma (ACC) [3]. These methylation changes can result in aberrant activation of oncogenes (by hypomethylation) or silencing of tumor suppressor genes (by hypermethylation). Several methylation-regulated, ACC-associated candidate genes have been identified, including *PTEN* [4], cyclin-dependent kinase inhibitors [5], *RASSF1*, *RARbeta2* [6] *p16^INK4a^*, *DAPK* [7], 14-3-3 sigma [8], E-cadherin [9], and *AQP1* [10]. Since DNA methylation and transcription regulation are frequent events in human cancers, our group has developed epigenomic screening methods to search for novel hypomethylated oncogene candidates in various types of human cancers, including salivary gland ACC [10].

Salivary gland ACC is a rare cancer, accounting for only 1% of all head and neck malignancies. Salivary gland ACC is well known for its neurotropic features, including frequent perineural invasion and perineural spread [11], although the prognostic value of perineural invasion for predicting survival is still contradictory. Salivary gland ACC is also prone to distant metastasis, which is seen in >40% of ACC patients [12]. Several promising candidate genes have been proposed that may help to elucidate the molecular mechanisms underlying distant metastasis [13], [14], [15], [16], [17], [18], [19], [20], [21]. Although expression profiling in primary ACC tumor samples provides convincing evidence in these studies, the majority of these works also utilized ACC cancer cell lines ACCM, ACC2, or ACC3; concern has been raised about contamination of these cell lines in a recent report [22].

Suprabasin (*SBSN*) was originally discovered in the suprabasal layers of stratified epithelium in the stomach and tongue [23] and was thought to play a role in epidermal differentiation [24]. Recently, our group employed an integrated, genome-wide screening technique to identify epigenetically-silenced oncogene candidates in non-small cell lung carcinoma (NSCLC) [25]. We found that *SBSN*, a novel oncogene candidate in NSCLC, plays an important role in promoting carcinogenesis.

In the current study, we investigated the role of *SBSN* in salivary gland ACC. We found that *SBSN* is important in maintaining a strong invasive/metastatic potential in ACC tumor cells. Furthermore, *SBSN* is also important in maintaining anchorage-dependent and anchorage-independent cell growth in ACC tumor cells. Moreover, the expression of *SBSN* is upregulated by CpG island demethylation, and hypomethylation of *SBSN* is significant in ACC versus normal salivary gland tissues (p<0.0001).

## Results

### Expression of *SBSN* is Upregulated by CpG Island Demethylation in SACC83

**Figure 1:**
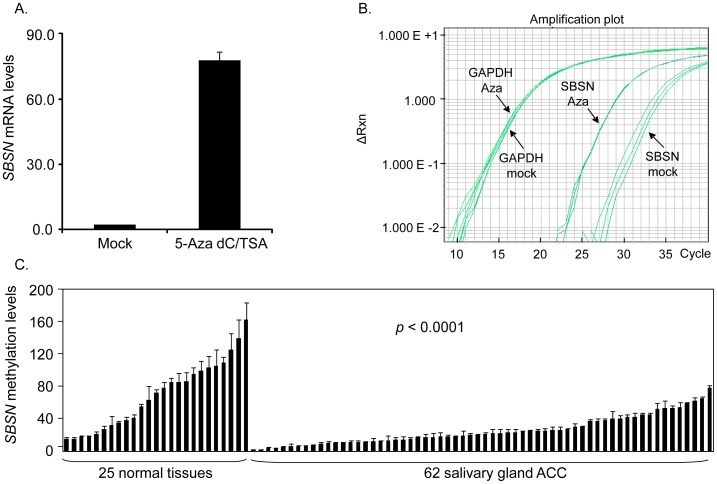
*SBSN* is hypomethylated in primary ACC samples, and its expression is induced by CpG island demethylation in SACC83. A. We first confirmed 5-aza-dC/TSA-induced expression of *SBSN* in SACC83 mRNA levels by qRT-PCR. B. Graph of actual data using TaqMan qRT-PCR analysis. X-axis, amplification cycle numbers; y-axis, ΔRn values used to plot signal attributable to the 5′ nuclease reaction, which reflects the quantity of amplicon. *SBSN* was amplified between 25–30 cycles in 5-aza-dC/TSA treatment, whereas it was amplified between 30–35 cycles in mock treatment. 5-aza-dC/TSA- or mock-treated samples were loaded in the same amount as indicated by *GAPDH*. C. qMSP was conducted in a paraffin-embedded ACC cohort, which consisted of 62 ACC samples and 25 normal salivary gland tissues. Significant hypomethylation in *SBSN* was shown in ACC versus normal salivary gland tissue (p<0.0001, Student’s t-test). *SBSN* methylation scores were normalized by β-actin. Error bars indicate the standard deviation.

We first confirmed that re-expression of *SBSN* was due to demethylation in SACC83, the only available ACC cell line to us. SACC83 was treated with 5-aza-2′-deoxycytidine plus trichostatin A (5-aza-dC/TSA) or mock reagent, and the expression levels of *SBSN* were then determined by quantitative reverse transcription PCR (qRT-PCR). *SBSN* was amplified 39.4±1.2-fold more (Fig. 1A), or 5.3 cycles earlier (Fig. 1B), with 5-aza-dC/TSA treatment than that in mock treatment, with a similar amount of input cDNA as shown by *GAPDH* (Fig. 1B).

### *SBSN* is Hypomethylated in Primary ACC Versus Normal Salivary Gland Tissues

We first analyzed DNA methylation levels of both *SBSN* CpG islands in a small ACC cohort consisting of eight ACC samples and eight normal salivary gland tissues by bisulfite genomic sequencing. *SBSN* hypomethylation was detected in six out of eight ACC, compared to two out of eight normal tissues (Fig. S1.). The *SBSN* CpG island that displayed greater differences in methylation between ACC and control tissues was a 102-bp region spanning nt 2858–2959 (relative to the transcription starting site), located in the second intron of *SBSN*. Quantitative methylation-specific PCR (qMSP) primers and probe were designed within this CpG island. With this set of qMSP primers and probe, we further analyzed the DNA methylation levels of *SBSN* in an ACC cohort consisting of 62 ACC samples and 25 normal salivary gland tissues. The clinical information of this cohort is summarized in Table 1. Our results show that *SBSN* methylation levels in ACC samples had an average value of 25.1±17.2 (range 2.1–78.4; median 20.4), while normal samples had an average value of 65.7±42.6 (range 13.4–162.1: median 63.9). *SBSN* was also significantly hypomethylated in ACC versus normal salivary gland tissues (*p*<0.0001) (Fig. 1C).

**Table 1:** Clinical and pathologic characteristics of patient populations.

Category	Subcategory	Normal	ACC
Patients, n		25	62
Age in years, median (range)		56 (39–76)	56 (17–86)
Sex, n (%)	Male	15 (60%)	40 (64.5%)
	Female	10 (40%)	22 (35.5%)
Smoking status, n (%)	No	9 (36%)	30 (48.4%)
	Yes	13 (52%)	24 (38.7%)
	Unknown	3 (12%)	8 (12.9%)
Tumor location, n (%)	Major salivary gland	–	33 (53.2%)
	Minor salivary gland	–	29 (46.8%)
Stage at diagnosis, n (%)	I	–	5 (8.1%)
	II	–	16 (25.8%)
	III	–	12 (19.4%)
	IV	–	18 (29.0%)
	Unknown	–	11 (17.7%)
Perineural invasion, n (%)	Positive	–	30 (48.4%)
	Negative	–	4 (6.5%)
	Not recorded	–	28 (45.2%)
Local recurrence, n (%)	Yes	–	17 (27.4%)
	No	–	41 (66.1%)
	Unknown	–	4 (6.5%)
Regional recurrence, n (%)	Yes	–	3 (4.8%)
	No	–	54 (87.1%)
	Unknown	–	5 (8.1%)
Distant metastasis, n (%)	Yes*	–	17 (27.4%)
	No	–	40 (64.5%)
	Unknown	–	5 (8.1%)
Overall survival in months, median (range)		–	59.3 (1.0–299.7)

* Time to distant metastasis ranged from 9.1–225.7 months, with a median of 48.1 months.

### *SBSN* is Important in Maintaining Anchorage-independent and Anchorage-Dependent Growth in SACC83

**Figure 2:**
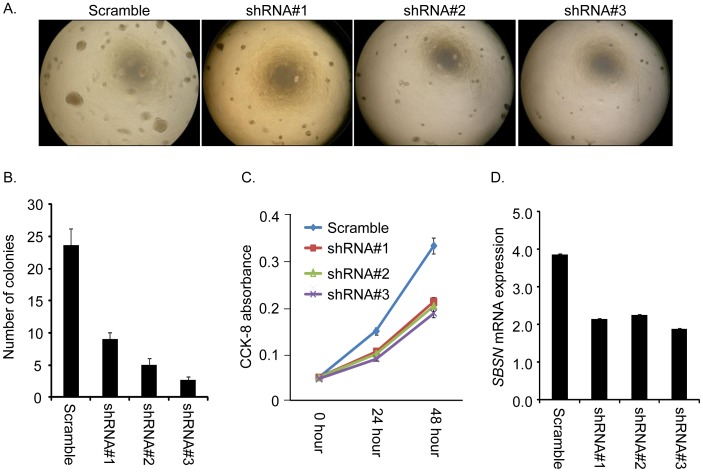
*SBSN* is important in maintaining anchorage-independent and anchorage-dependent growth in SACC83. Scramble, shRNA 1, shRNA 2, and shRNA 3 indicate the control and three types of *SBSN* shRNAs used to establish stable clones in SACC83. A, Representative photographs of anchorage-independent growth by soft agar assay. The size of colonies indicates the focus formation ability of each type of stable clone. B, The number of colonies counted in each type of stable clone. C, Anchorage-dependent cell proliferation assay. CCK-8 absorbance indicates the amount of cells at time-points 0, 24, and 48 hours. Scramble clones grew faster than *SBSN* shRNA stable clones at 24 and 48 hours, p<0.01. Statistical comparisons were performed with Student’s t-test. Error bars indicate the standard deviation of triplicate assays. D. *SBSN* mRNA levels in different stable clones were determined by qRT-PCR. This confirmed that *SBSN* was silenced by ∼50% in shRNAs clones compared to scramble clones.

In order to evaluate the functions of *SBSN* in ACC carcinogenesis, we established stable clones of SACC83 with three different *SBSN* shRNAs and one scramble shRNA as a control. To prove that *SBSN* plays a role in anchorage-independent focus formation in SACC83, we used soft agar analysis. Our data demonstrate that only scramble shRNA clones showed large focus formation, while all three *SBSN* shRNA stable clones had formation of foci that were noticeably smaller in size. Representative photographs of the colony focus formation assay are shown in Fig. 2A. The number of foci formed for each type of shRNA stable clone is shown in Fig. 2B. To prove that *SBSN* plays a role in anchorage-dependent cellular proliferation, we studied cell proliferation rates by plating different types of shRNA clones onto conventional 6-well plates and then counting the amount of cells at 0, 24, and 48 hours using the CCK8 assay. Our results show that the scramble clone grew significantly faster than all three *SBSN* shRNA clones at 24 and 48 hours, p<0.01 (Fig. 2C). Finally, the *SBSN* mRNA levels of each type of shRNA stable clone were confirmed by qRT-PCR: *SBSN* was silenced by ∼50% compared to scramble clones, as shown in Fig. 2D.

### *SBSN* is Important in Maintaining the Invasive and Metastatic Capability in SACC83

**Figure 3:**
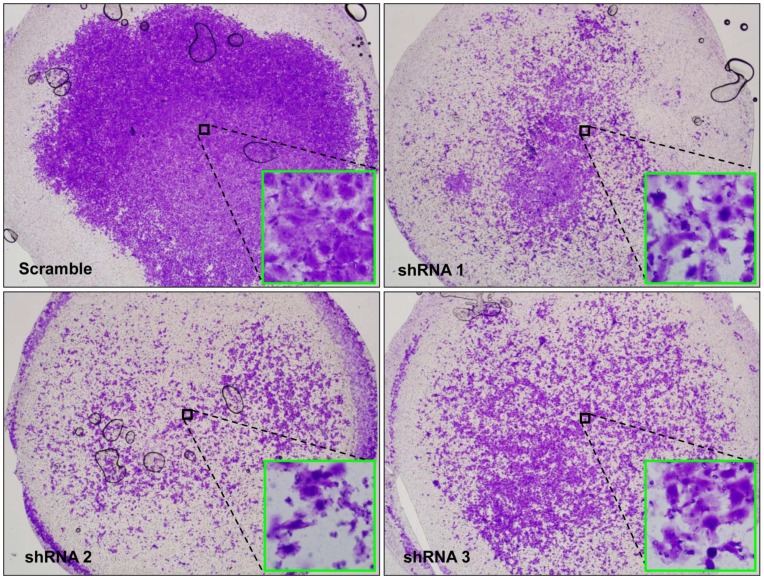
*SBSN* is important in maintaining invasion and metastatic capability in SACC83. Matrigel invasion assay was performed with scramble control and three types of *SBSN* shRNA stable clones made from SACC83, as indicated by scramble, shRNA 1, shRNA 2, and shRNA 3. Representative photos of whole transwell membranes are pictured at 4× magnification; the inset pictures were taken at 20× magnification at randomly selected central locations. This metastasis analysis was performed in triplicate.

Because perineural invasion and distant metastasis are characteristic features of ACC, we further studied the role of *SBSN* in invasion and metastasis by the *in vitro* Matrigel invasion assay. We observed that, for all three *SBSN* shRNA clones, a significantly decreased number of cells migrated through the 8-µm pore transwell membrane, compared to the scramble clone (Fig. 3). The metastatic patterns of cells were not evenly distributed throughout the membrane; therefore, the overall view (4X) and randomly selected subregions (inset, 20X) are shown (Fig. 3).

### Clinical Correlation of *SBSN* Methylation Status

We next sought to evaluate possible associations between *SBSN* methylation levels and clinical variables (see Table S1). *SBSN* methylation levels were not correlated with gender, age, primary site, smoking history, tumor (T) stage, nodal (N) stage, presence or absence of metastasis (M stage) at diagnosis, perineural invasion, surgical margin status, or development of local recurrence or distant metastasis. *SBSN* hypomethylation was associated with a trend towards increased risk of developing regional recurrence, but this result did not reach statistical significance (p = 0.10, Fisher exact test). Additionally, *SBSN* methylation levels were not associated with overall survival or time to disease relapse.

## Discussion

It is well known that both distant metastasis and perineural invasion are prominent clinical features of ACC [11], [12]. Distant metastasis is seen in >40% of ACC patients. Perineural invasion is thought to be one reason for local recurrence after seemingly adequate surgical excision. These factors suggest that ACC tumor cells have a unique potential for migration locally and distantly.

With the functional work performed in this study, *SBSN*-silenced stable cell line clones demonstrated a significantly reduced invasive/metastatic capability, anchorage-dependent growth, and anchorage-independent growth compared to scramble control clones. Our data therefore suggest that *SBSN* plays an important role in maintaining the invasive and metastatic properties of ACC. These discoveries are consistent with a recent study conducted by Formolo *et al* [26]. They compared the secretome profiles of glioblastoma cell lines with high versus low invasive potential, as assessed with the Matrigel invasion assay, and showed that *SBSN* is one of the candidate proteins that are responsible for the highly invasive capability of glioblastoma multiforme (GBM). Their *SBSN* results in GBM are of special interest for ACC because such findings may help to explain the neurotropism that ACC exhibits. In addition, our lab [25] recently reported that *SBSN* played an oncogenic role in the carcinogenesis of non-small cell lung cancer (NSCLC). Taking these data together, *SBSN* might be a novel oncogene candidate relevant in ACC.

It is widely accepted that methylation changes in the CpG island(s) overlapping the promoter region can regulate the transcription of both oncogenes and tumor suppressor genes in human cancer. Recently, DNA methylation changes in regions that do not overlap with promoters have also been suggested to play regulatory roles in gene transcription [27], [28]. These regions are termed differential methylation regions, or DMR. This new knowledge suggests that DNA methylation can regulate transcription from more flexible sites than previously defined. In the current study, the *SBSN* CpG island that showed the most differential methylation was a 102-bp region spanning nt 2858–2959 (relative to the transcription starting site, TSS), located in the second intron of *SBSN*. Our results show that *SBSN* CpG island hypomethylation induced *SBSN* transcription after 5-Aza dC/TSA treatment in cell lines, and *SBSN* was significantly hypomethylated in primary salivary gland ACC versus normal tissues. We did observe considerable overlap of the methylation values of *SBSN* in normal and tumor tissues. This finding might suggest a dynamic variability in DNA methylation changes during carcinogenesis. With our current experimental design, it is impossible to accurately delineate the quantitative relationship between *SBSN* methylation levels to *SBSN* transcriptional levels. These limitations are due to lack of good quality primary tumor RNA for qRT-PCR validation analysis and a lack of *SBSN* antibodies for immunostaining analysis. Additionally, there are no mature quantitative techniques to evaluate the underlying relationship between DNA methylation and transcription.

Interestingly, the *SBSN* CpG island identified overlaps a binding motif of a transcription factor, BORIS (Brother of the Regulator of Imprinted Sites). BORIS is paralogous to CTCF [29], functions as a transcriptional regulator, and forms methylation-dependent insulators [30]. Our recent studies demonstrate that the expression of *SBSN* was upregulated by DNA hypomethylation changes in this CpG island, and the hypomethylation within this region was induced by BORIS binding [31]. Given the methylation differences that were detected, *SBSN* hypomethylation within this BORIS binding site might be a promising biomarker for salivary gland ACC. This is of important clinical significance because patient DNA can be obtained by non-invasive methods, and DNA methylation changes can be reliably detected and quantified by highly efficient techniques like qMSP [32].

In summary, *SBSN* is a novel oncogene candidate in ACC and plays an important role in the carcinogenesis and metastasis of salivary gland ACC. *SBSN* hypomethylation may potentially be a useful non-invasive marker for ACC.

## Materials and Methods

### Ethics Statement

Primary ACC tissue was obtained via the Johns Hopkins Pathology Department under a Johns Hopkins Institutional Review Board-approved protocol.

### Clinical Samples

For the paraffin-embedded samples, 62 blocks with high tumor yield were selected after additional confirmation of ACC histology by an experienced head and neck pathologist (JAB). Eight 10-micron slides were cut, and the tumors were manually microdissected to yield at least 80% tumor purity. Normal parotid tissue samples that had been paraffin-embedded were also used, after histologic confirmation that no tumor or inflammation was contained within those slides and that the tissue used was distant from separate benign lesions.

DNA extraction from paraffin slides was performed as described previously [10], [33]. Briefly, samples were digested in 1% SDS and 50 µg/mL proteinase K (Roche Applied Science, Indianapolis, IN) at 48°C overnight. DNA was then purified by phenol–chloroform extraction and ethanol precipitation. The DNA was subsequently resuspended in LoTE (EDTA 2.5 mmol/L and Tris–HCl 10 mmol/L) and stored at −80°C until use.

### Cell Lines

The ACC cell line SACC83 was cultured at RPMI with 1% penicillin/streptomycin and 10% FBS and grown in a 37°C incubator with 5% CO_2_.

### Quantitative Reverse Transcription PCR (qRT-PCR)

Total RNA was measured and adjusted to the same amount for each sample, and cDNA synthesis was performed using the qScript cDNA Synthesis Kit (Quanta BioSciences, Gaithersburg, MD). The final cDNA products were used as the templates for subsequent qRT-PCR analysis. TaqMan gene expression assays with premixed primers and probe were ordered from AB Applied Biosystems (Carlsbad, CA), the catalog numbers are Hs01078781_m1* for *SBSN* and Hs99999905_m1 for *GAPDH*. QRT-PCR was carried out according to the manufacturer’s instructions. The conditions were 95°C for 5 minutes, followed by 40 cycles of 95°C for 15 seconds and 64°C for 1 minute. *GAPDH* was examined to ensure accurate relative quantitation for qRT-PCR.

### 5-aza-2′-deoxycytidine Plus Trichostatin A Treatment

We treated SACC83 in triplicate with 5-aza-2′-deoxycytidine (5-aza-dC, Sigma-Aldrich) and trichostatin A (TSA, Sigma-Aldrich, St. Louis, MO) as described previously [33], [34]. Briefly, cells were treated in triplicate with fresh 5 uM 5-Aza dC every 24 hours for a total of 96 hours and with 300 nmol/L TSA for the last 24 hours. Baseline expression was established by mock-treated cells with the same volume of blank controls. Total cellular RNA was isolated using the RNeasy kit (Qiagen, Valencia, CA) according to the manufacturer’s instructions.

### Bisulfite Treatment and Bisulfite Genomic Sequencing

The EpiTect Bisulfite Kit (Qiagen, Valencia, CA) was used to convert unmethylated cytosines in genomic DNA to uracil, according to the manufacturer’s instructions [33]. Converted DNA was stored at −80°C until use. Subsequently, bisulfite-treated DNA was amplified with primers designed using MethPrimer to span areas of CpG island(s) [35]. Primer sequences, specifically designed to contain no CG dinucleotides, were: forward 5′- TTT TTT AGG TTT TAT GAG GGG TTT T -3′ and reverse 5′- TAC TAT TAC CAA CCC CAA ATC CTA C -3′. Touch-down PCR was performed, and products were purified using the QIAquick 96 PCR Purification Kit (Qiagen, Valencia, CA), according to the manufacturer’s instructions [33]. Purified PCR products were then subjected to direct sequencing by the Johns Hopkins Sequencing Core.

### Quantitative Methylation-specific PCR (qMSP)

QMSP conditions and data interpretation were described previously [10], [36]. Leukocyte DNA from a healthy individual was methylated *in vitro* with excess *Sss*I methyltransferase (New England Biolabs, Ipswich, MA) to generate completely methylated DNA, and serial dilutions (90-0.009 ng) of this bisulfite-treated DNA were used to construct a calibration curve for each plate. All samples were within the range of sensitivity and reproducibility of the assay based on the amplification of the internal reference standard (threshold cycle value for β-actin of 40). The relative level of methylated DNA in each sample was determined as a ratio of qMSP–amplified gene to β-actin (reference gene) and then multiplied by 100 for easier tabulation (average value of triplicates of the gene of interest divided by the average value of triplicates of β-actin × 100). The *SBSN* primer sequences were: forward 5′- TGG TTT AGA CGT CGA AGT TT -3′, and reverse 5′- CTA CAA CCT ACC GTA CCC G -3′. The *SBSN* probe was 6FAM 5′- ACG CCG TTC CTC CCC ACC CA -3′TAMRA. QMSP was carried out using the following conditions: 95°C for 5 minutes, followed by 45 cycles at 95°C for 15 seconds and 60°C for 1 minute.

### Silencing *SBSN* in Cell Lines, Cell Proliferation, Soft Agar and Metastasis Assays

*SBSN* shRNA and corresponding scramble shRNA were ordered from Origene (Rockville, MD), catalog number TG301828. LipoD293™ (SignaGen Laboratories, Gaithersburg, MD) was used for transfection according to the manufacturer’s recommendations. Transfected cells were cultured in medium with 0.6 ug/ml puromycin (Invitrogen, Grand Island, NY) for two weeks to establish stable clones before proceeding to functional analyses.

For the cell proliferation assay, cells transfected with *SBSN* shRNAs and scramble shRNA were measured at 0, 24, and 48 hours using the Cell Counting Kit-8 (CCK-8) (Dojindo, Rockville, MD) as described previously [10]. Absorbance (450 nm minus 650 nm) was measured by the Spectramax M2e 96-well fluorescence plate reader (Molecular Devices, Sunnyvale, CA). All growth experiments were performed in triplicate for all cell lines and vectors.

For the soft agar assay (anchorage-independent), stably transfected clones were counted, and approximately 2000 cells were added to each well of a 6-well plate. The bottom layer was composed of 0.5% agar in RPMI1640, while the cells were suspended in the top layer of 0.3% agar in RPMI1640. Soft agar assays were cultured in medium with 0.6 µg/ml puromycin and incubated at 37°C for 8–12 days before data analysis.

To assess the migration/invasion ability of cells transfected with *SBSN* shRNAs and scramble shRNA, Matrigel invasion assays were performed using 8-µm pore filter inserts in 24-well plates (Sigma-Aldrich, St. Louis, MO) coated with Matrigel (BD Biosciences, Sparks, MD). Cells were trypsinized, washed with PBS three times, and re-suspended in serum-free RPMI 1640. A total of 100,000 cells were plated in each well. Chemoattractant media with FBS (500 µl) was added to the lower wells. After 24 hours of incubation at 37°C in a CO_2_ incubator, the inserts were removed. Cells on the upper surface that had not invaded the membrane were removed with a cotton swab. The cells on the lower surface of the membrane were fixed and stained in thiazine and eosin solution using Diff-Quik II solution (Dade Behring, Marburg, Germany) and were sealed on the slides. Migrated cells were photographed by microscopy at 4× and 20× magnification. Each clone was analyzed in triplicate.

### Clinical Correlation Analysis

All variables were summarized using descriptive statistics. The median *SBSN* methylation level was calculated and used to divide the data set into two groups, with methylation values above or below the median. Differences in the distributions of categorical variables between the two groups were assessed using chi-squared or Fisher exact tests. For ordinal variables, two-sample Wilcoxon rank-sum tests were used to evaluate the hypothesis that patients above and below the median methylation level were from populations with the same distribution. Continuous variables were summarized using means and ranges, and differences between the two groups were compared using two-sided t-tests. Differences were considered statistically significant if p was less than 0.05. All analyses were performed using Stata 11.0 software (StataCorp, College Station, TX). Survival curves were calculated with the method of Kaplan and Meier and compared using the log-rank statistic. Overall survival was calculated from the date of diagnosis to the day of death or last follow-up. Time to disease relapse was calculated from the date of diagnosis to the day of local or regional recurrence, metastasis, or death. Computations for survival analyses were performed using SAS 9.2 (Cary, NC).

## Supporting Information

Figure S1 **Bisulfite sequencing results of SBSN in 8 normal (N1–N8) and 8 adenoid cystic carcinoma (T1–T8) samples.** In the region depicted, 6/8 of the tumors demonstrated hypomethylation, as indicated by the bisulfite conversion of the CG site to a TG, while only 2/8 normal samples showed hypomethylation at a single CG site.
(TIF)

Table S1 **Clinical and outcome measures of patients with high and low** ***SBSN*** **methylation levels.**
(TIFF)
