# Supplementary figures and images for: Suprabasin Is Hypomethylated and Associated with Metastasis in Salivary Adenoid Cystic Carcinoma
Source: PLoS One. 2012 Nov 7;7(11):e48582. doi: 10.1371/journal.pone.0048582 (PMC3492451; doi:10.1371/journal.pone.0048582)

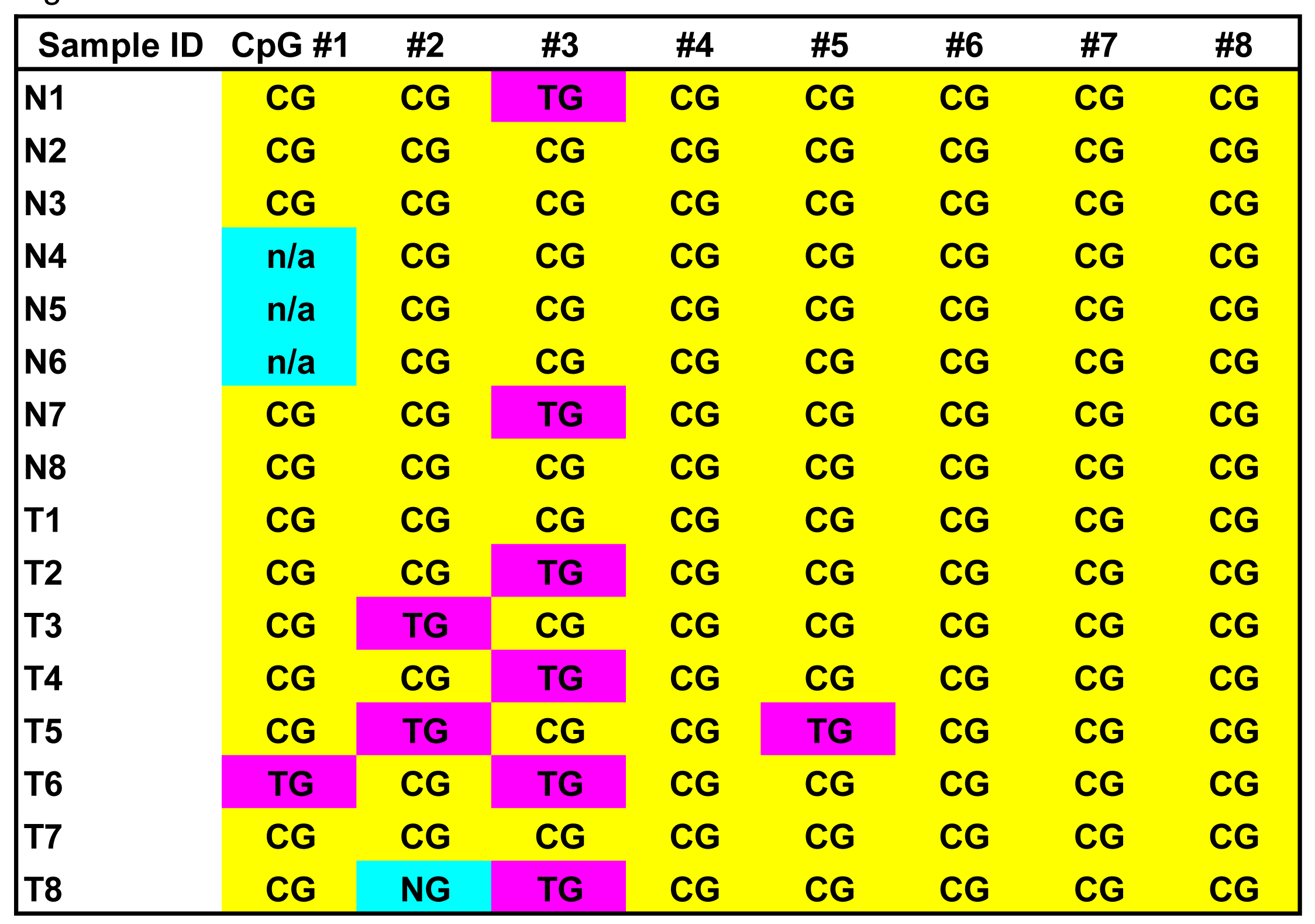

Supplement: Figure S1 — Bisulfite sequencing results of SBSN in 8 normal (N1–N8) and 8 adenoid cystic carcinoma (T1–T8) samples. In the region depicted, 6/8 of the tumors demonstrated hypomethylation, as indicated by the bisulfite conversion of the CG site to a TG, while only 2/8 normal samples showed hypomethylation at a single CG site. (TIF) [file pone.0048582.s001.tif]
